# Investigation of Small Bowel Abnormalities in HIV-Infected Patients Using Capsule Endoscopy

**DOI:** 10.1155/2017/1932647

**Published:** 2017-03-20

**Authors:** Eiji Sakai, Takuma Higurashi, Hidenori Ohkubo, Kuhihiro Hosono, Atsuhisa Ueda, Nobuyuki Matsuhashi, Atsushi Nakajima

**Affiliations:** ^1^Department of Gastroenterology and Hepatology, Yokohama City University School of Medicine, Yokohama, Japan; ^2^Department of Gastroenterology, NTT Medical Center Tokyo, Tokyo, Japan; ^3^Department of Hematology and Clinical Immunology, Yokohama City University School of Medicine, Yokohama, Japan

## Abstract

HIV infection is reportedly associated with an increased permeability of the intestinal epithelium and can cause HIV enteropathy, which occurs independently of opportunistic infections. However, the characteristics of small bowel abnormalities attributable to HIV infection are rarely investigated. In the present study, we assessed the intestinal mucosal changes found in HIV-infected patients and compared them with the mucosa of healthy control subjects using capsule endoscopy (CE). Three of the 27 HIV-infected patients harbored gastrointestinal opportunistic infections and were thus excluded from subsequent analyses. The endoscopic findings of CE in HIV-infected patients were significantly higher than those in control subjects (55% versus 10%, *P* = 0.002); however, most lesions, such as red spots or tiny erosions, were unlikely to cause abdominal symptoms. After validating the efficacy of CE for the diagnosis of villous atrophy, we found that the prevalence of villous atrophy was 54% (13/24) among HIV-infected patients. Interestingly, villous atrophy persisted in patients receiving long-term antiretroviral therapy, though most of them exhibited reconstituted peripheral blood CD4+ T cells. Although we could not draw any conclusions regarding the development of small bowel abnormalities in HIV-infected patients, our results may provide some insight regarding the pathogenesis of HIV enteropathy.

## 1. Introduction

The management of opportunistic infections of the gastrointestinal tract is crucial for improving the morbidity and mortality rates of AIDS patients. Since the introduction of highly active antiretroviral therapy (HAART), the frequency of opportunistic infections has been substantially reduced [1]. Meanwhile, HIV itself has been regarded as a mediator of small bowel enteropathy. As the lymphoid tissue of the gut plays an important role in the defense against external pathogens, the gastrointestinal mucosa can become the main target of HIV infection [2, 3]. In addition, the function of the intestinal epithelial barrier is closely associated with progressive HIV replication [4]. Previous reports have suggested that intestinal mucosal barrier defects occur independently of opportunistic infections [5–7], reflecting the impact of HIV infection itself. Therefore, early gastrointestinal mucosal events should be carefully examined to better understand the pathogenesis of HIV infection. Crypt hyperproliferation and villous shortening, resulting in partial villous atrophy, reportedly occur as specific morphological features of HIV enteropathy and can be observed at all stages of HIV infection [8–10]. However, most investigations have only examined the duodenum, because of the difficulty in accessing the small bowel. Therefore, small intestinal abnormalities attributed to HIV infection remain poorly characterized.

Capsule endoscopy (CE) was first introduced in 2000 [11] and has since become established as a useful modality for diagnosing small bowel abnormalities [12–15]. CE enables the entire small bowel to be visualized at a magnification in a minimally invasive manner. Although CE is predominantly used for patients with obscure gastrointestinal bleeding (OGIB), its usefulness has also been demonstrated in patients with celiac disease (CD), which is an immune-mediated disorder occurring in people genetically susceptible to gluten [16]. Because villous atrophy is frequently observed in both CD and HIV-infected patients, we speculated that CE examination might be useful for revealing the characteristics of HIV enteropathy.

Since the present study was conducted to reveal mucosal changes attributed to HIV infection itself, we first performed an entire gastrointestinal endoscopic examination and excluded patients with specific opportunistic gastrointestinal infectious diseases. Subsequently, we confirmed the validity of the application of CE for the diagnosis of villous atrophy in HIV-infected patients. The characteristics of small bowel abnormalities were compared between HIV-infected patients and healthy control subjects. In addition, we investigated the correlation between clinical parameters related to HIV infection and small bowel abnormalities. Our results will provide insight into the details of HIV enteropathy.

## 2. Material and Methods

### 2.1. Patients

Between May 2007 and October 2014, 27 consecutive HIV-infected patients who underwent CE at Yokohama City University Hospital were enrolled in this study. All of the patients had undergone upper and lower endoscopic examinations prior to the CE. As this study aimed to reveal mucosal changes attributable to HIV infection itself, patients with specific opportunistic infectious diseases (e.g., infection with cytomegalovirus (CMV), mycobacteriosis, cryptosporidium, or tuberculosis) were excluded. In addition, a fecal culture was performed to exclude bacterial enteritis (e.g., *Salmonella* spp., *Escherichia coli*, and *Campylobacter* spp.). Moreover, patients using aspirin and/or nonsteroidal anti-inflammatory drugs were excluded, because such drugs can induce small bowel injury [13, 15]. A total of 21 healthy adult subjects were also included as a control group for the comparison of small bowel abnormalities. We registered the patient data, including the age, sex, smoking history, alcohol history, hemoglobin concentration, and albumin and CRP values. Clinical symptoms (abdominal pain, diarrhea, and gastrointestinal bleeding) and the details of HIV infection (history of antiretroviral therapy, follow-up duration, viral load, and peripheral blood CD4 count) were also evaluated at the time of the initial CE. The antiretroviral therapy consisted of a standard combination of two nucleoside reverse transcriptase inhibitors together with either a nonnucleoside reverse transcriptase inhibitor or a protease inhibitor (HAART). The study protocol was approved by the Ethics Committee of Yokohama City University Hospital. Written informed consent was obtained from all of the subjects prior to their participation in the study.

### 2.2. Capsule Endoscopy

The protocol for the CE procedure has been previously reported [13, 14]. In summary, the patients were instructed to swallow the CE capsule (PillCam SB/SB2; Given Imaging, Yoqneam, Israel) in a solution of dimethicone after fasting overnight, with no other bowel preparation. They were allowed to drink clear liquids 2 hours after they had swallowed the capsule and to eat a light meal 4 hours after. After 8 hours, we confirmed whether the capsule had passed through the ileocecal valve using the Real-Time Viewer (Given Imaging) and the examination was continued to achieve complete entire small bowel visualization. Two CE experts (with experience evaluating more than 150 CE videos) separately read and interpreted the complete CE videos. When discrepancies in interpretation occurred, both experts reviewed the findings simultaneously and reached a consensus. Each of the CE videos was divided into two segments of equal length according to the small-bowel transit time: the first segment was considered to represent the proximal small bowel, and the second to represent the distal small bowel.

### 2.3. Definition of Small Bowel Villous Atrophy

The duodenal mucosal pathology was evaluated by an experienced pathologist and was used as the gold standard for the diagnosis of villous atrophy. At least 4 biopsies were performed in the lower duodenum. The specimens were cut longitudinally at 4 *μ*m and stained with hematoxylin and eosin. According to the modified Marsh classification [17], grade ≥ 3 was regarded as being positive for villous atrophy. Villous atrophy was endoscopically diagnosed as positive when the following features were found using CE in the duodenum: reduction or absence of Kerckring's folds, mosaic mucosal pattern, and scalloping [16]. To validate the application of CE to the diagnosis of villous atrophy, the sensitivity, specificity, and positive predictive value were evaluated. In addition, the time until the appearance of the first villi and time with features of atrophy were also recorded, expressed in hours and fraction of hours [18].

### 2.4. Outcomes after CE Examination

For patients with persisting symptoms in which the CE examination identified severe significant abnormalities, a subsequent balloon-assisted endoscopy (BAE) was performed as a therapeutic intervention or for diagnostic biopsy, where necessary. To reveal the association between villous atrophy and HIV infection, the expression of intestinal CD4+ T cells was examined using duodenal specimens and immunohistochemistry staining (NCL-L-CD4-368; Novocastra, Newcastle, United Kingdom).

### 2.5. Statistical Analysis

All the data were presented as the mean ± standard deviation, unless otherwise specified. The statistical significances of the differences in the values of the clinical parameters were evaluated using Fisher's exact test and an unpaired Student's *t*-test. The *P* value was 2 sided, and *P* < 0.05 was used to determine statistical significance. All the analyses were performed using the SPSS, ver. 11.0 (SPSS Inc., Chicago IL, USA).

## 3. Results

### 3.1. Characteristics of Enrolled Patients

The demographic and clinical characteristics of the enrolled patients are shown in Table 1. Most of the HIV-infected patients were males (96%) and were significantly older than the control subjects (45.0 ± 10.2 years versus 32.2 ± 4.3 years, *P* < 0.001). As for clinical symptoms, diarrhea was commonly present in HIV-infected patients (63% versus 0%, *P* < 0.001). Among the 27 HIV-infected patients, 18 (67%) had received HAART and their viral loads were suppressed below the limit of detection. Meanwhile, 6 (22%) patients underwent CE at the time of the initial diagnosis of HIV infection. The median follow-up duration after the initial diagnosis was 4.0 years (range, 0–20 years). An entire small bowel examination was successfully achieved in most of the case, while the CE capsule failed to reach the cecum in only one case with CMV enteritis.

### 3.2. HIV-Infected Patients with Specific Opportunistic Infections

A subsequent BAE and pathological examination revealed that three of the 27 HIV-infected patients harbored gastrointestinal opportunistic infections. One patient with OGIB who exhibited multiple small bowel ulcers was diagnosed as having CMV-induced small bowel enteritis and began receiving ganciclovir therapy. The other patients with chronic diarrhea were diagnosed as having either jejunal Kaposi's sarcoma or small bowel mycobacteriosis. These patients had been untreated and therefore subsequently received HAART after the opportunistic infections had been controlled. As this study aimed to reveal the mucosal changes attributable to HIV infection itself, these patients were excluded. Finally, a total of 24 HIV-infected patients were eligible for the subsequent analyses.

### 3.3. Validity of the Diagnosis for the Villous Atrophy Using CE

**Figure 1:**
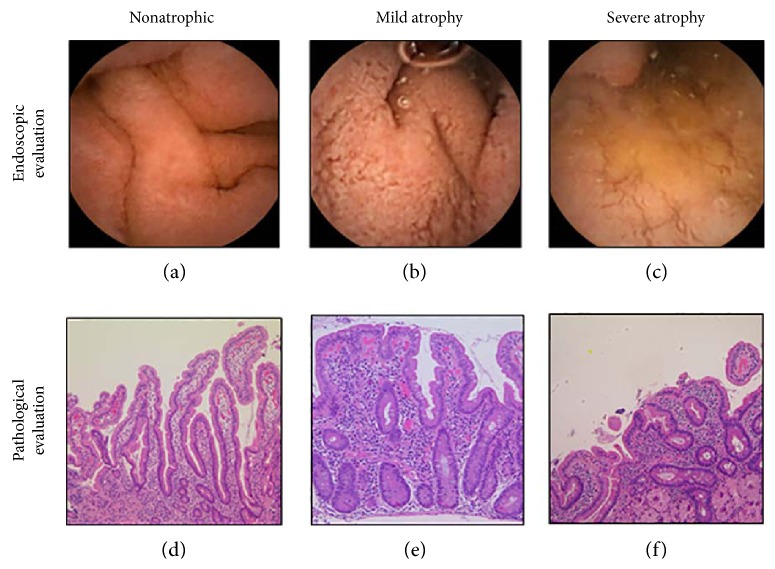
Representative images of villous atrophy. Villous atrophy was endoscopically diagnosed as positive when the reduction or absence of Kerckring's folds, a mosaic mucosal pattern and scalloping, was confirmed in the duodenum (a–c). A biopsy was performed at the lower duodenum, and villous atrophy was evaluated using the modified Marsh classification [17] (d–f). Marsh stage ≥ 3 was diagnosed as villous atrophy positive. (a) Nonatrophic villi. (b) Reduction of villi. (c) Mosaic pattern of mucosa. (d) Marsh 0. (e) Marsh 3a. (f) Marsh 3c.

Representative images of villous atrophy are presented in Figure 1. Of the 15 HIV-infected patients who underwent a histopathological evaluation of the duodenal mucosa, endoscopic markers of villous atrophy were identified in 47% (7/15) of the cases. When the pathological diagnosis was used as the gold standard, the sensitivity, specificity, and positive predictive value for the diagnosis of villous atrophy using CE were 100%, 89%, and 86%, respectively (Table 2).

### 3.4. Small Bowel Abnormalities Identified in HIV-Infected Patients

The comparison of small bowel abnormalities between HIV-infected patients and control subjects is summarized in Table 3. The diagnostic yield of CE in HIV-infected patients was significantly higher than that among the control subjects (55% versus 10%, *P* = 0.002). The prevalence and number of erosions were also significantly higher among HIV-infected patients (42% versus 5%, *P* = 0.01, and 0.8 ± 0.6 versus 0.1 ± 0.1, *P* = 0.02), while ulceration was rarely identified in both groups (13% versus 0%, *P* = 0.24). There were no significant differences in the distributions of small bowel abnormalities. Other significant abnormalities, such as angioectasia and tumors, were not identified in either group. The prevalence and number of small bowel abnormalities were not different between patients with and those without a peripheral blood CD4 count < 200/*μ*L (71% versus 47%, *P* = 0.39, and 1.5 ± 1.5 versus 1.0 ± 1.4, *P* = 0.50).

Since the efficacy of the endoscopic diagnosis of villous atrophy was demonstrated, we assessed the prevalence of villous atrophy among all the enrolled patients. Although no endoscopic markers of villous atrophy were found in the control subjects, markers were identified in 54% (13/24) of the HIV-infected patients. A reduction or absence of Kerckring's folds was frequently observed (11/13, 85%), while a mosaic mucosal pattern and scalloping were rarely identified (2/13, 15%). Among 13 HIV-infected patients with villous atrophy, the time to the appearance of first villi was 0.3 ± 0.6 hours. The mean time with features of villous atrophy was 0.5 ± 0.4 hours. When the distribution of the villous atrophy was evaluated, the proximal small bowel mucosa was predominantly involved (21% versus 6%).

### 3.5. Association between HIV Infection and Villous Atrophy

A comparison of the clinical parameters according to the presence of villous atrophy is presented in Table 4. There were no significant differences in the hemoglobin concentration or the serum albumin and/or CRP values. Diarrhea was frequently present in patients with villous atrophy, but the significance was borderline (77% versus 36%, *P* = 0.10). Antiretroviral therapy was equally used in both villous atrophy positive and negative patients (77% and 73%, *P* = 0.81). There was no significant difference in the presence of villous atrophy when compared between patients with and without a peripheral blood CD4 count < 200/*μ*L (38% versus 18%, *P* = 0.28). In addition, the intestinal CD4+ cell expression level was also similar.

## 4. Discussion

In the present study, we investigated the details of small bowel abnormalities in HIV-infected patients using CE. Based on the results of entire gastrointestinal endoscopic examinations and subsequent pathological evaluations, we identified three (11%) cases of opportunistic infectious disease. These cases occurred in symptomatic naïve HIV-infected patients, thus necessary to doubt harboring opportunistic infections. Importantly, all the infectious foci were limited to the small bowel, which would have made early detection difficult even after a conventional gastrointestinal endoscopic examination. Moreover, Mönkemüller et al. reported that approximately 10% of HIV-infected patients taking HAART can present with gastrointestinal opportunistic infections [19]. Therefore, the application of CE should be carefully considered, if the possible presence of gastrointestinal opportunistic infections is suspected.

Oette et al. investigated gastrointestinal abnormalities in HIV-infected patients and reported that a number of findings with therapeutic implications were identified in the small bowel [20]. Of note, most of their patients were markedly immunosuppressed (peripheral blood CD4 count < 200/*μ*L); therefore, the effects of the HIV infection itself could not be discussed. Unlike their report, we included patients whose viral replication had been intensively suppressed using HAART (67%); therefore, only a few findings with therapeutic implications were identified. Active HIV replication leads to a local increase in inflammatory cytokine, such as TNF*α*, and impairs mucosal barrier function [21]. The degree of inflammation within the gut is reportedly correlated with viral replication [22]. Epple et al. reported that the suppression of HIV replication by HAART improved mucosal barrier defects [23]. Although we confirmed a significantly higher diagnostic yield of CE among HIV-infected patients, most of the small bowel abnormalities identified in whom seem less relevant (e.g., red spots and/or tiny erosions). In addition, we confirmed that the presence of small bowel injuries did not differ between treated and untreated HIV patients. Taken together, we consider that external pathogens are probably required to cause significant small bowel damages, though the mucosal barrier defect itself can be attributed to HIV infection.

Previous reports have indicated a potential role for CE as a diagnostic tool for villous atrophy [16, 24]. In a recent meta-analysis assessing the accuracy of CE as a diagnostic tool in CD, the sensitivity of this modality was 89%, with a specificity of 95% [25]. In addition, CE is reportedly useful for accessing the therapeutic response in CD patients [26]. Our results suggested that CE might also be useful for assessing the presence of villous atrophy in HIV-infected patients. We revealed that the proximal small bowel was likely to present villous atrophy. Although the distribution of villous atrophy was similar to CD patients [16], the levels of villous atrophy seemed comparatively low among the HIV-infected patients, since a scalloping and/or mosaic pattern (which is often observed in CD patients) was rarely identified. Consistent with our report, Troeger et al. also confirmed that severe villous atrophy is rarely seen in HIV-infected patients, despite the resemblance of the histological changes in small bowel structures [27]. This finding probably reflects the fact that most HIV-infected patients continue to have a good nutritional condition, despite having a high frequency of villous atrophy.

Gastrointestinal symptoms reportedly improve soon after the introduction of HAART [28], suggesting that HAART can ameliorate enteropathy. However, we confirmed that 36% of the patients treated with HAART still presented with diarrhea. Although other causes of diarrhea (e.g., drug-induced) should be ruled out, we believe that the presence of diarrhea might be partly associated with villous atrophy. Importantly, villous atrophy was still observed in patients who had received long-term HAART, though most of them exhibited reconstituted peripheral blood CD4+ T cells. Meanwhile, HIV-infected patients rarely exhibit a reconstituted CD4+ T cell population in the small bowel, possibly because viral replication continues at a low level, even after the introduction of HAART [29]. Guadalupe et al. reported that genes involved in inflammation and stress were upregulated in such patients, contrary to the decreased expression of genes involved in digestive and absorptive functions [30]. These mucosal conditions are thought to help the sustained transformation of the intestinal mucosa, resulting in partial villous atrophy. Unfortunately, we could not confirm an association between villous atrophy and intestinal CD4+ T cell expression, probably because of the limited number of available samples. On the other hand, Batman et al. reported that HAART can restore normal crypt structure by inhibiting HIV-driven stem cell hyperproliferation at the crypt bases [31]. To confirm the pathogenesis of HIV enteropathy, additional investigations, such as flow cytometry and/or western blot analyses, are needed. In addition, multifocal small bowel biopsies may help to understand the pathogenesis of small bowel abnormalities, including villous atrophy and mucosal injuries.

The present study had several limitations. First, we lacked adequate control patients with persistent symptoms and instead used nonsymptomatic healthy subjects. In addition, male patients were predominantly enrolled in this study. These factors could potentially affect the validity of our findings. Second, the overall number of patients included in the study was comparatively small. Third, despite the clear correlation between pathological and endoscopic findings obtained from distal duodenum, we could not assess the specimen obtained from middle to distal small bowel. Fourth, we did not perform the follow-up CE examinations, which could have limited our conclusions. Finally, unspecific CE findings may not be regarded as HIV-associated disease, though we tried to exclude patients with external gastrointestinal infectious diseases and/or drug-induced small bowel injury. Further studies are needed to clarify the relevance of the spectrum of pathological results.

## 5. Conclusions

Our results indicated that HIV infection itself can induce small bowel mucosal injury; however, such injury is unlikely to cause clinical symptoms. In addition, we also revealed that HIV infection is closely associated with villous atrophy, which is not completely restored even after HAART. Although we could not make any conclusions regarding the pathogenesis of small bowel abnormalities in HIV-infected patients, previous reports highlight the importance of mucosal barrier impairment caused by HIV infection itself. Since the gastrointestinal mucosa is the main target of HIV infection, CE examination may help to better understand the pathogenesis of HIV infection.

## Conflicts of Interest

The authors declare that there is no conflict of interest regarding the publication of this paper.

## Figures and Tables

**Table 1:** Characteristics of enrolled patients.

	HIV-infected patients	Control subjects	*P* value
Number	27	21	
Sex			
Male	26 (96%)	19 (90%)	0.57
Female	1 (4%)	2 (10%)	
Age, mean ± SD, year	45.0 ± 10.2	32.2 ± 4.3	<0.001
Smoking habit	9 (33%)	6 (29%)	0.76
Alcohol intake	8 (30%)	8 (38%)	0.56
Clinical symptoms			
Abdominal pain	2 (7%)	0 (0%)	0.50
Diarrhea	16 (63%)	0 (0%)	<0.001
Gastrointestinal bleeding	2 (7%)	0 (0%)	0.50
Duration after initial diagnosis, median (range), year	4.0 (0–20)	n.a.	n.a.
HAART	18 (67%)	n.a.	n.a.

HAART: highly active antienteroviral therapy; *P* values were analyzed using Fisher's exact test or Student's *t*-test for age.

**Table 2:** Correlation between endoscopic and pathological findings of small bowel atrophy.

	Capsule endoscopy evaluation	Pathological evaluation
Atrophy positive	7	6
Atrophy negative	8	9

The sensitivity, specificity, and positive predictive value for the diagnosis of villous atrophy using capsule endoscopy were 100%, 89%, and 86%, respectively.

**Table 3:** Comparison of small bowel abnormalities diagnosed by capsule endoscopy.

	HIV-infected patients	Control subjects	*P* value
Number	24	21	
Diagnostic yield, *N* (%)	13 (55%)	2 (10%)	0.002
Redness, prevalence, *N* (%)	7 (29%)	2 (10%)	0.14
Total, *N* (mean)	10 (0.4)	3 (0.1)	0.17
Proximal, *N* (mean)	5 (0.2)	0	0.06
Distal, *N* (mean)	5 (0.2)	3 (0.1)	0.63
Erosion, prevalence, *N* (%)	10 (42%)	1 (5%)	0.01
Total, *N* (mean)	18 (0.8)	2 (0.1)	0.02
Proximal, *N* (mean)	11 (0.5)	1 (0.1)	0.01
Distal, *N* (mean)	7 (0.3)	1 (0.1)	0.19
Ulceration, prevalence, *N* (%)	3 (13%)	0	0.24
Total number, *N* (%)	4 (0.2)	0	0.10
Proximal, *N* (mean)	2 (0.1)	0	0.36
Distal, *N* (mean)	2 (0.1)	0	0.36
Villous atrophy, *N* (%)	13 (54%)	0	<0.001

Among 27 HIV-infected patients, three patients diagnosed as having Kaposi's sarcoma, small bowel mycobacteriosis, and cytomegarovirus-induced small bowel enteritis, respectively, were excluded from subsequent analyses. Each of the CE videos was divided into two segments of equal length according to the small-bowel transit time; the first segment was considered as representing the proximal small bowel, and the second as representing the distal small bowel. *P* values were analyzed using Fisher's exact test or Student's *t*-test.

**Table 4:** Association between clinical factors and villous atrophy.

	Villous atrophy (+)	Villous atrophy (−)	*P* value
Number	13	11	
Age, year	43.8 ± 11.1	44.9 ± 8.8	0.80
Duration after initial diagnosis, median (range), year	4.0 (0–9.0)	4.0 (0–20.0)	0.35
Clinical symptoms			
Abdominal pain	1 (8%)	0 (0%)	>0.99
Diarrhea	10 (77%)	4 (36%)	0.10
Serum albumin value, g/dL	4.4 ± 0.5	4.4 ± 0.6	0.89
Hemoglobin value, g/dL	13.8 ± 1.9	14.1 ± 1.5	0.34
CRP, mg/dL	0.2 ± 0.3	0.2 ± 0.3	0.65
HAART therapy	10 (77%)	8 (73%)	0.81
Peripheral blood CD4 count < 200/*μ*L	5 (38%)	2 (18%)	0.28

HAART: highly active antienteroviral therapy; *P* values were analyzed using Fisher's exact test or Student's *t*-test for age and the Mann-Whitney *U* test for follow-up duration.

## References

[1] Murphy E. L., Collier A. C., Kalish L. A. (2001). Highly active antiretroviral therapy decreases mortality and morbidity in patients with advanced HIV disease. *Annals of Internal Medicine*.

[2] Mattapallil J. J., Douek D. C., Hill B., Nishimura Y., Martin M., Roederer M. (2005). Massive infection and loss of memory CD4+ T cells in multiple tissues during acute SIV infection. *Nature*.

[3] Guadalupe M., Reay E., Sankaran S. (2003). Severe CD4+ T-cell depletion in gut lymphoid tissue during primary human immunodeficiency virus type 1 infection and substantial delay in restoration following highly active antiretroviral therapy. *Journal of Virology*.

[4] Epple H. J., Allers K., Tröger H. (2010). Acute HIV infection induces mucosal infiltration with CD4+ and CD8+ T cells, epithelial apoptosis, and a mucosal barrier defect. *Gastroenterology*.

[5] Greenson J. K., Belitsos P. C., Yardley J. H., Bartlett J. G. (1991). AIDS enteropathy: occult enteric infections and duodenal mucosal alterations in chronic diarrhea. *Annals of Internal Medicine*.

[6] Stockmann M., Fromm M., Schmitz H., Schmidt W., Riecken E. O., Schulzke J. D. (1998). Duodenal biopsies of HIV-infected patients with diarrhoea exhibit epithelial barrier defects but no active secretion. *AIDS*.

[7] Keating J., Bjarnason I., Somasundaram S. (1995). Intestinal absorptive capacity, intestinal permeability and jejunal histology in HIV and their relation to diarrhoea. *Gut*.

[8] Ullrich R., Riecken E. O., Zeitz M. (1991). Human immunodeficiency virus-induced enteropathy. *Immunologic Research*.

[9] Batman P. A., Miller A. R., Forster S. M., Harris J. R., Pinching A. J., Griffin G. E. (1989). Jejunal enteropathy associated with human immunodeficiency virus infection: quantitative histology. *Journal of Clinical Pathology*.

[10] Ullrich R., Zeitz M., Heise W., L'age M., Höffken G., Riecken E. O. (1989). Small intestinal structure and function in patients infected with human immunodeficiency virus (HIV): evidence for HIV-induced enteropathy. *Annals of Internal Medicine*.

[11] Iddan G., Meron G., Glukhovsky A., Swain P. (2000). Wireless capsule endoscopy. *Nature*.

[12] Rastogi A., Schoen R. E., Slivka A. (2004). Diagnostic yield and clinical outcomes of capsule endoscopy. *Gastrointestinal Endoscopy*.

[13] Sakai E., Endo H., Taniguchi L. (2013). Factors predicting the presence of small bowel lesions in patients with obscure gastrointestinal bleeding. *Digestive Endoscopy*.

[14] Sakai E., Endo H., Taguri M. (2014). Frequency and risk factors for rebleeding events in patients with small bowel angioectasia. *BMC Gastroenterology*.

[15] Endo H., Sakai E., Taniguchi L. (2014). Risk factors for small-bowel mucosal breaks in chronic low-dose aspirin users: data from a prospective multicenter capsule endoscopy registry. *Gastrointestinal Endoscopy*.

[16] Collin P., Rondonotti E., Lundin K. E. (2012). Video capsule endoscopy in celiac disease: current clinical practice. *Journal of Digestive Diseases*.

[17] Oberhuber G., Granditsch G., Vogelsang H. (1999). The histopathology of coeliac disease: time for a standardized report scheme for pathologists. *European Journal of Gastroenterology & Hepatology*.

[18] Atlas D. S., Rubio-Tapia A., Van Dyke C. T., Lahr B. D., Murray J. A. (2011). Capsule endoscopy in nonresponsive celiac disease. *Gastrointestinal Endoscopy*.

[19] Mönkemüller K. E., Lazenby A. J., Lee D. H., Loudon R., Wilcox C. M. (2005). Occurrence of gastrointestinal opportunistic disorders in AIDS despite the use of highly active antiretroviral therapy. *Digestive Diseases and Sciences*.

[20] Oette M., Stelzer A., Göbels K. (2009). Wireless capsule endoscopy for the detection of small bowel diseases in HIV-1-infected patients. *European Journal of Medical Research*.

[21] Stockmann M., Schmitz H., Fromm M. (2000). Mechanisms of epithelial barrier impairment in HIV infection. *Annals of the New York Academy of Sciences*.

[22] Kotler D. P., Reka S., Clayton F. (1993). Intestinal mucosal inflammation associated with human immunodeficiency virus infection. *Digestive Diseases and Sciences*.

[23] Epple H. J., Schneider T., Troeger H. (2009). Impairment of the intestinal barrier is evident in untreated but absent in suppressively treated HIV-infected patients. *Gut*.

[24] DeGaetani M., Tennyson C. A., Lebwohl B. (2013). Villous atrophy and negative celiac serology: a diagnostic and therapeutic dilemma. *The American Journal of Gastroenterology*.

[25] Rokkas T., Niv Y. (2012). The role of video capsule endoscopy in the diagnosis of celiac disease: a meta-analysis. *European Journal of Gastroenterology & Hepatology*.

[26] Murray J. A., Rubio-Tapia A., Van Dyke C. T. (2008). Mucosal atrophy in celiac disease: extent of involvement, correlation with clinical presentation, and response to treatment. *Clinical Gastroenterology and Hepatology*.

[27] Troeger H., Loddenkemper C., Schneider T. (2009). Structural and functional changes of the duodenum in human norovirus infection. *Gut*.

[28] Kotler D. P., Shimada T., Snow G. (1998). Effect of combination antiretroviral therapy upon rectal mucosal HIV RNA burden and mononuclear cell apoptosis. *AIDS*.

[29] Brenchley J. M., Douek D. C. (2008). HIV infection and the gastrointestinal immune system. *Mucosal Immunology*.

[30] Guadalupe M., Sankaran S., George M. D. (2006). Viral suppression and immune restoration in the gastrointestinal mucosa of human immunodeficiency virus type 1-infected patients initiating therapy during primary or chronic infection. *Journal of Virology*.

[31] Batman P. A., Kapembwa M. S., Belmonte L. (2014). HIV enteropathy: HAART reduces HIV-induced stem cell hyperproliferation and crypt hypertrophy to normal in jejunal mucosa. *Journal of Clinical Pathology*.

